# Microsatellite instability in penile cancer: comparative analysis of primary tumors and metastases

**DOI:** 10.3389/fruro.2026.1908537

**Published:** 2026-08-26

**Authors:** Hadson Silva Araujo, Gustavo Ramos Teixeira, Ana Carolina Laus, Gustavo Noriz Berardinelli, Daniel D Almeida Preto, Rui Manuel Reis, Flavio Mavignier Cárcano

**Affiliations:** 1 Department of Medical Oncology, Barretos Cancer Hospital, Barretos, Brazil; 2 Molecular Oncology Research Center, Barretos Cancer Hospital, Barretos, Brazil; 3 Barretos School of Health Sciences Dr. Paulo Prata – FACISB, Barretos, Brazil; 4 Pathology Department, Barretos Cancer Hospital, Barretos, Brazil; 5 Life and Health Sciences Research Institute (ICVS), Medical School, University of Minho, Braga, Portugal; 6 3ICVS/3B’s-PT Government Associate Laboratory, Braga, Portugal; 7 Oncoclinicas & Co - Medica Scientia Innovation Research (MEDSIR), São Paulo, Brazil; 8 Faculdade de Ciências Médicas de Minas Gerais, Belo Horizonte, Brazil

**Keywords:** HPV, immunohistochemistry, lymph node metastasis, microsatellite instability, p16, penile cancer

## Abstract

**Background:**

Penile cancer (PeCA) presents high morbidity and mortality and is more prevalent in underdeveloped countries. The presence of nodal metastasis at diagnosis or as early recurrence carries a worse prognosis, making it important to determine whether clinically relevant biomarkers are maintained between primary tumors and corresponding lymph node metastases. We aim to describe the presence of DNA microsatellite instability (MSI) in primary PeCA tumors and locoregional lymph nodes affected by metastatic cells, as well as to assess HPV status through p16 expression.

**Methods:**

A total of 116 patients were comparatively evaluated between the primary tumor and lymph node metastases. The evaluation of p16 and mismatch repair proteins was done by immunohistochemistry, and MSI status was assessed using a hexa-plex marker by polymerase chain reaction, followed by fragment analysis.

**Results:**

All patients underwent a standard lymphadenectomy (inguinal or pelvic), with a pN0 frequency of 33.6%. MSI-H was found in three patients (2.6%), with correspondence between the presence of the biomarker in the primary tumors and the lymph node metastases. A second validation was performed using IHC for MMR, with MLH1/PMS2 loss in MSI-H cases. 22.8% of patients expressed p16 by immunohistochemistry. p16 positivity was associated with a 55% reduction in the risk of death. All MSI-H patients were p16 positive.

**Conclusion:**

MSI-H occurs in 2,6% of the sample and is associated predominantly with MLH1/PMS2 loss and p16 positivity. Together, these findings suggest potential interactions between HPV- associated carcinogenesis and genomic instability. Further prospective, biomarker-driven trials are warranted to define their prognostic and therapeutic relevance in PeCA.

## 1 Introduction

Penile cancer (PeCA) is a rare disease, yet, in developing countries, the incidence increases, and in Brazil, the incidence varies between 2.9 - 6.8/100,000 inhabitants (1) and 3–4 per 100,000 inhabitants in Uganda (2). The GLOBOCAN estimate for 2025, 39885 new cases, leading to 14485 deaths (3). The etiological factors associated with PeCA are phimosis, genital warts, HPV and HIV infection, the presence of lichen sclerosus and UV light to treat psoriasis (4–8). Epidermoid carcinoma is the most prevalent histology in PeCA (95%). In 2022, the World Health Organization (WHO) published the new international classification of PeCA: they are currently divided into HPV-associated and HPV-independent (9).

PeCA spreads via the lymphatic route, initially through the inguinal lymph nodes, followed by the pelvic and retroperitoneal lymph nodes (10). The presence of lymph node metastasis is therefore the main factor in poor prognosis (11). Another well-described factor that corroborates histopathological stratification is the presence of HPV DNA, represented by the expression of p16, and is correlated with a 54% reduction in the risk of mortality and a 37% reduction in the risk of disease recurrence, compared to the absence of HPV DNA (12). The combined prevalence of HPV DNA is 50.8% among all patients with PeCA; the predominant oncogenic HPV type was HPV16 with 68.3%, followed by HPV6 and HPV18 with 8.1% and 6.7%, respectively (13).

Although cancer treatment is increasingly guided by molecular biomarkers, their concordance between primary penile tumors and corresponding lymph node metastases remains poorly understood in PeCA. Immunotherapy offers new treatment opportunities for many cancer subtypes (14). Microsatellite instability-high (MSI-H) is a predictive biomarker of response to immunotherapy. The mismatch repair (MMR) system corrects potential mutations that occur during DNA replication. DNA microsatellite regions are subject to replication errors, and in the event of a deficiency in the repair system (dMMR), these errors can lead to instability in these regions (15, 16). The mutational tumor burden (TMB) is also used as a surrogate for the use of immunotherapy, and the correlation between TMB and MSI-H has been demonstrated in a real-world study, regardless of tumor type or treatment line (17). A study of 72 PeCA patients using immunohistochemistry revealed one patient with dMMR (deficient MMR0 and 12 with pMMR (proficient MMR). Of the 13 cases tested, one was MSI-H, with no correlation observed with the dMMR case (18). Another PeCA study with a larger sample size (n = 397) showed that 15% of cases were classified as high TMB (10 mut/Mb or more) (19).

Herein, we aimed to compare MSI status between primary tumors and corresponding lymph node metastases in a representative cohort of patients with PeCA. We also assessed HPV status using p16 as a surrogate marker and correlated these findings with clinicopathological characteristics.

## 2 Patients and methods

### 2.1 Study population and selection of cases

We retrospectively included consecutive patients diagnosed with penile squamous cell carcinoma between 2010 and 2023 at Barretos Cancer Hospital who underwent surgical treatment and had available formalin-fixed paraffin-embedded (FFPE) tissue from the primary tumor and lymph node metastasis. Patients lacking sufficient biological material for molecular or immunohistochemical analyses were excluded. Clinical-pathological features were retrieved from the medical records and collected using REDCap electronic data capture tool (20). Among the 116 cases, 114 patients had samples available for molecular and immunohistochemistry analysis; two patients were excluded due to a lack of biological material. All patients underwent inguinal or pelvic lymphadenectomy, and the decision to perform surgery was in accordance with the protocols of the local urology department. The analysis was made comparatively between the primary tumor and the lymph node metastases. This study was approved by the local IRB under Protocol No. CAAE 53660821.0.0005437.

### 2.2 p16 Immunohistochemistry

Immunohistochemistry (IHC) was performed on paraffin-embedded tissue from the patients’ tumors, in sections up to 4 μm thick, using the BenchMark^®^ Ventana Ultra automated staining system (Roche Diagnostics, Switzerland). p16 expression was assessed using the Cintecp16 Histology 50 Kit (CE) from Roche Diagnostics for automated immunohistochemistry, when not expressed, they are classified by the WHO as HPV-independent, and when positive, HPV-associated. Samples with strong and diffuse nuclear and cytoplasmic staining in more than 75% of the tumor cells were considered positive for p16 (21, 22).

### 2.3 Mismatch repair Immunohistochemistry

For cases in which MSI-H was detected by PCR, immunohistochemistry for four MMR proteins (MLH1, PMS2, MSH2, and MSH6) was performed. The antibody clones used were MLH1 (clone ES05, Agilent Technologies Canada Inc.), MSH2 (clone G219-1129, Becton Dickinson Biosciences, Canada), MSH6 (clone EPR3945, Abcam, Canada), and PMS2 (clone A16-4, BD Biosciences). Normal adjacent tissue from each sample served as a positive control. MMR protein loss was defined by the absence of IHC staining in the tumor cell nucleus, while normal cells remained stained, ensuring the technical validity of the experiment. The immunohistochemical staining results were evaluated according to the scoring system reported in the literature: (i) MMR proficient (pMMR): cases with all four MMR stains positive; (ii) dMMR: cases with loss of one of the two heterodimers, including MLH1/PMS2 or MSH2/MSH6 (23).

### 2.4 DNA isolation and microsatellite instability (MSI) assay

Tumor DNA was isolated from FFPE sections using QIAamp DNA Micro Kit (Qiagen, Germany), accordantly manufacturer’s guideline. The MSI assay was performed using HT-MSI+ kit (Cellco, São Carlos, Brazil) composed of six quasi-monomorphic mononucleotide repeat markers (NR27, NR21, NR24, BAT25, BAT26, and HSP110), following the manufacturer’s protocol (24, 25). The analysis was performed using 3500 Genetic Analyzer automated sequencer (Applied Biosystems, USA) and visualized by the GeneMapper software version 4 (Applied Biosystems, USA), according to manufacturer’s recommendations. Cases with the presence of two or more markers out of the quasimonomorphic variation range (QMVR) were classified as MSI-H (presence of microsatellite instability), and cases with one or without markers out of QMVR were classified as MSS (absence of microsatellite instability).

### 2.5 Statistical analysis

The comparative analysis methodology was done using the kappa index and the chi-squared test. Survival curves were plotted using the Kaplan–Meier method and the event of interest (death) was considered for the outcome of overall survival (OS). Alive patients and those lost to follow-up were censored. Univariate and multivariate analysis were performed using Cox regression method. Statistical analyses were performed using IBM SPSS Statistics for Mac OS, Version 25 (IBM). P values < 0.05 were considered statistically significant.

## 3 Results

### 3.1 Clinical–pathological characterization of PeCA patients

One hundred and sixteen cases were selected according to the inclusion and exclusion criteria (**Table 1**), two patients did not have enough samples from either the primary or the metastatic lymph nodes. All patients were squamous cell carcinomas (SCCs). The median age was 55 years. All patients underwent lymphadenectomy, either inguinal (91.4%), pelvic (0.8%) or both (7.8%). More than three quarters of the patients had less than an elementary school education. Of samples, 13.8% of them were pN1, 19.0% pN2 and 33.6% pN3. (**Table 1**).

**Table 1:** Clinical-pathological characteristics of patients with PeCA.

Variable	n (%)
Patients with PeCA	116 (100.0)
Age	
Mean	55
Min-max	23-86
Education level	
Illiterate	63 (54.3)
Incomplete primary school	21 (22.4)
Incomplete high school	8 (6.9)
Other	24 (16.4)
Smoking	
Yes	35 (30.2)
No	81 (69.8)
Postectomy	
Yes	12 (10.3)
No	104 (89.7)
Type of primary surgery	
Partial glandectomy	9 (7.8)
Partial penectomy	88 (75.9)
Total penectomy	15 (12.9)
Other	4 (3.4)
Type of lymphadenectomy	
Inguinal	106 (91.3)
Pelvic	1 (0.9)
Inguinal and pelvic	9 (7.8)
Neo/adjuvant treatment	
Yes	34 (29.3)
No	82 (70.7)
First-line chemotherapy	
Yes	22 (19.0)
No	94 (81.0)
Extra capsular extension	
Yes	47 (40.5)
No	69 (59.5)
Nodal pathological staging	
pN0	39 (33.6)
pN1	16 (13.8)
pN2	22 (19.0)
pN3	39 (33.6)
Staging	
0	1 (0.9)
I	4 (3.4)
II	33 (28.4)
III	37 (31.9)
IV	41 (35.4)
Histological subtype	
Usual	81 (71.0)
Verrucous	6 (5.3)
Sarcomatoid	1 (0.9)
Basaloid	26 (22.8)

PeCA, penile cancer.

**Figure 1:**
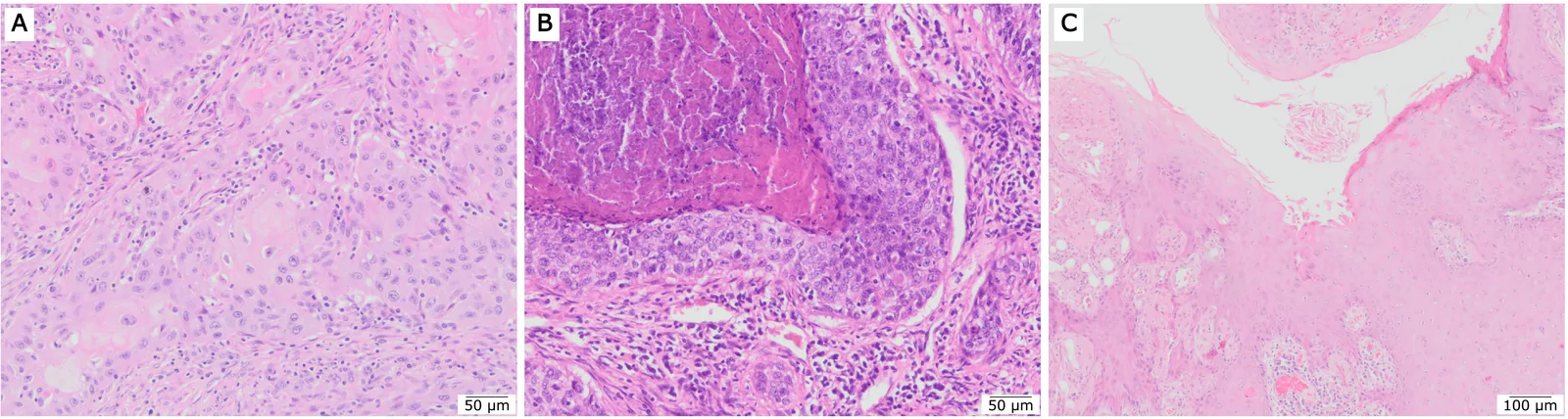
Micrographs of HE stain in PeCA tumor cells. **(A)** Usual (20x); **(B)** Basaloid (20x); **(C)** Verrucous (10x).

According to the WHO 2022, 71,0% of cases were the usual squamous cell carcinoma cell carcinoma histological subtype, 5.3% were verrucous, 0.8% sarcomatoid and 22,8% basaloid subtype (**Figure 1**).

Of note, 39 cases underwent lymphadenectomy and were classified as pN0, and therefore, metastasis analysis was carried out in 77 cases. Approximately 30% of patients received neo or adjuvant chemotherapy, and almost 20% received first-line chemotherapy. Patients received predominantly platinum-based combination chemotherapy according to institutional protocols. (**Table 1**).

### 3.2 p16 expression and histological analysis

**Figure 2:**
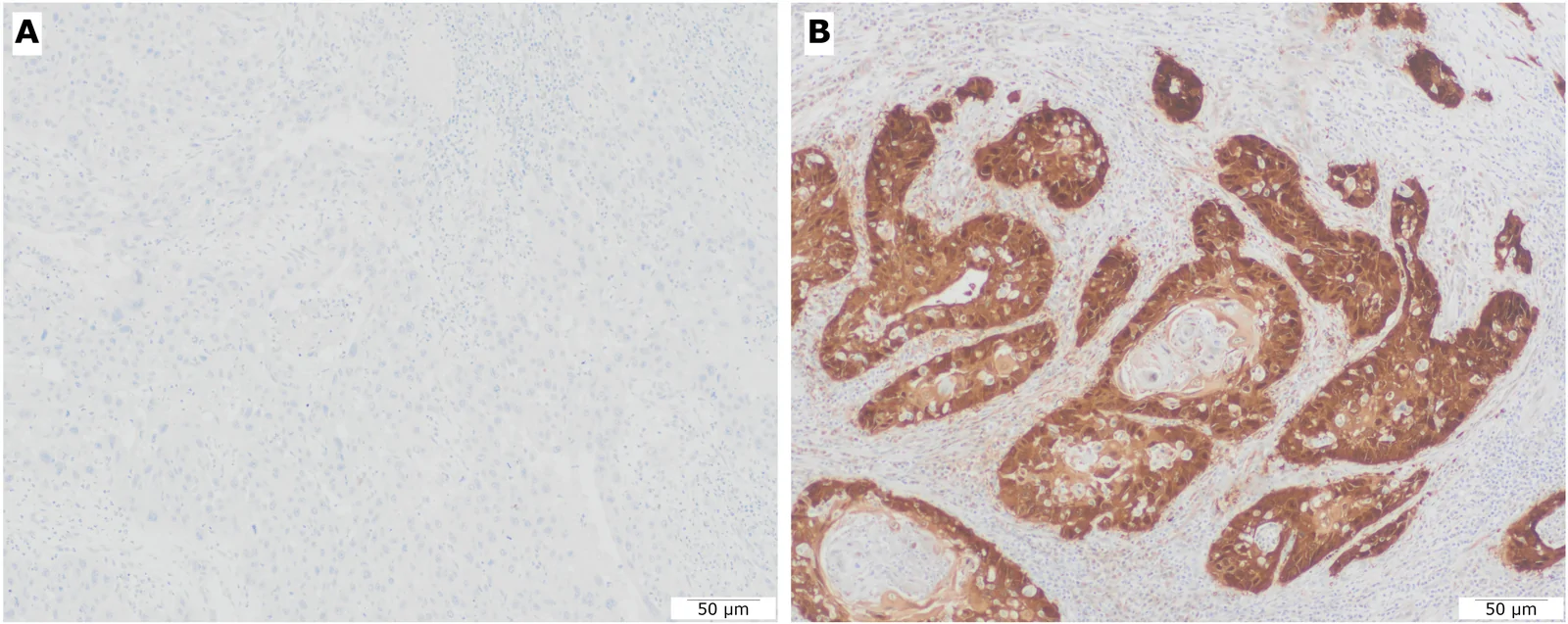
Micrographs of immunohistochemistry staining of p16 in PeCA tumor cells (20x). **(A)** p16 negative; **(B)** p16 positive.

**Figure 3:**
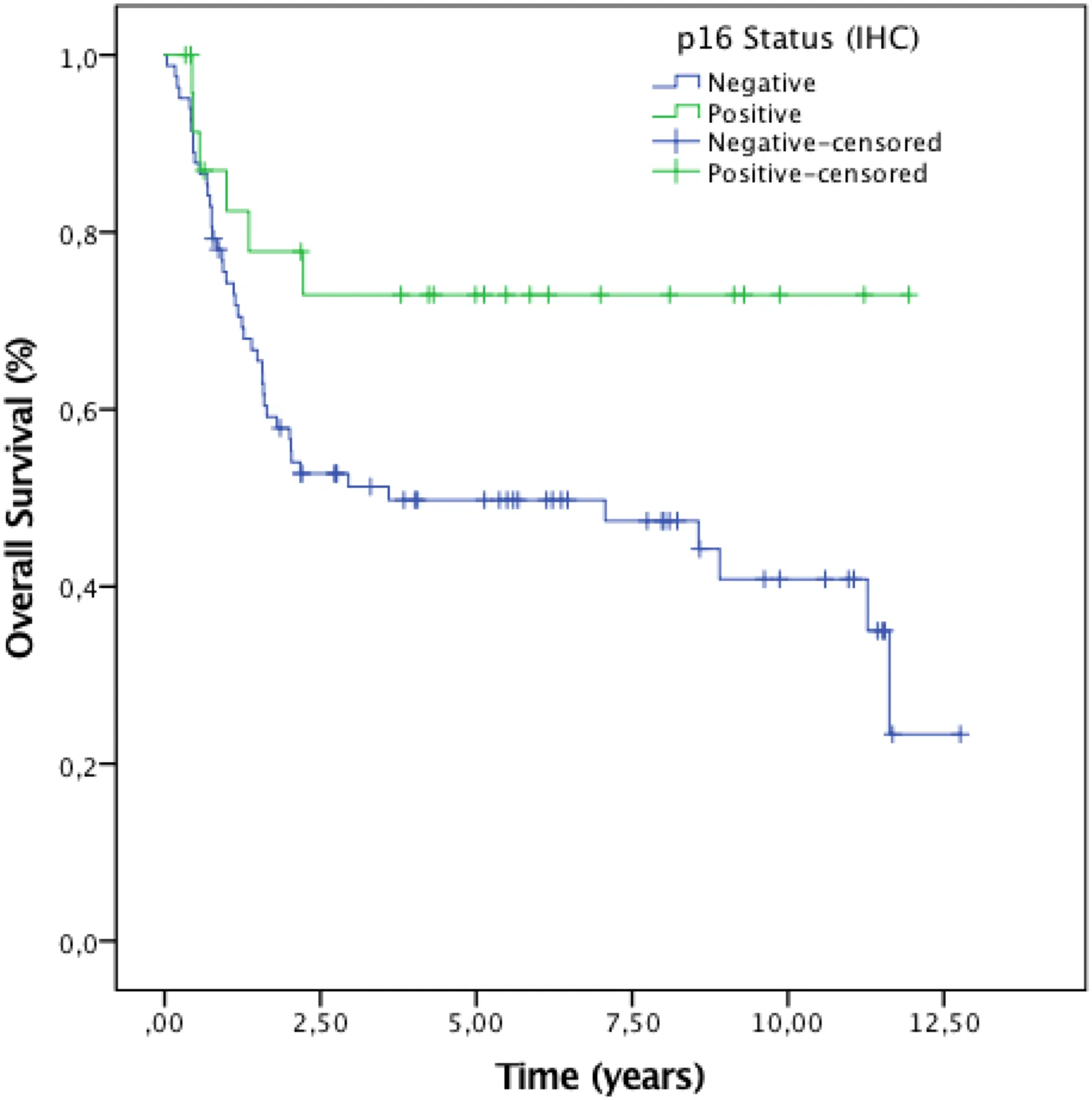
Kaplan–Meier overall survival curve according to p16 expression.

As above mentioned, 26 cases (22.8%) had positive p16 expression (**Figure 2**), being considered HPV-positive. This evaluation was performed on primary tumors in 103 cases, except when material was unavailable, in which case it was performed on metastases in 11 cases. The survival rate (**Figure 3**) of patients who expressed p16 did not reach the median, while patients who were p16 negative had a survival rate of 3.5 years (p 0.027). Univariate Cox regression analysis identified extracapsular extension, advanced nodal stage, AJCC stage III–IV, and receipt of chemotherapy as significant predictors of worse overall survival (**Table 2**). Although p16 expression was not significantly associated with survival in the univariate analysis, it showed a trend toward significance and was therefore included in the multivariable model. In the multivariable analysis, AJCC stage III–IV remained independently associated with an increased risk of death (HR 5.9), whereas positive p16 expression was independently associated with a reduced risk of death (HR 0.45) (**Table 2**).

**Table 2:** Univariate and multivariate analysis of clinicopathologic characteristics and survival in patients with PeCA.

		Univariate analysis			Multivariate analysis		
Variable	Category	HR	95%CI	p value	HR	95%CI	p value
Smoking	No (ref)	0.92	0.52-1.64	0.789			
	Yes						
Family history	No (ref)	0.88	0.49-1.55	0.66			
	Yes						
Previous postectomy	No (ref)	0.98	0.42-2.30	0.969			
	Yes						
Extra capsular extension	No (ref)	2.08	1.22-3.55	**0.007**			
	Yes						
Nodal staging	pN0/pN1 (ref)	2.93	1.66-5.18	**<0.001**			
	pN2/pN3						
Combined staging	I e II (ref)	5.6	2.53-12.61	**<0.001**	5.951	2.63-13.46	**0.001**
	III e IV						
Chemotherapy	No (ref)	2.2	1.29-3.82	**0.004**			
	Yes						
Expression of p16	No (ref)	0.5	0.23-1.06	0.074	0.45	0.21-0.96	**0.04**
	Yes						

HR, hazard ratio; CI, confidence interval; PeCA, penile cancer.Bold values means statistically significant.

### 3.3 Microsatellite instability analysis

MSI analysis of primary tumors was performed on 99 samples, of which 2 were MSI-H, 70 were MSS, and 27 were inconclusive due to DNA quality issues related to DNA degradation associated with formalin-fixed paraffin-embedded (FFPE) tissue in older archival specimens. The analysis of lymph node metastasis, 69 samples were analyzed, of which 3 were MSI-H, 55 MSS, and 11 were inconclusive.

**Figure 4:**
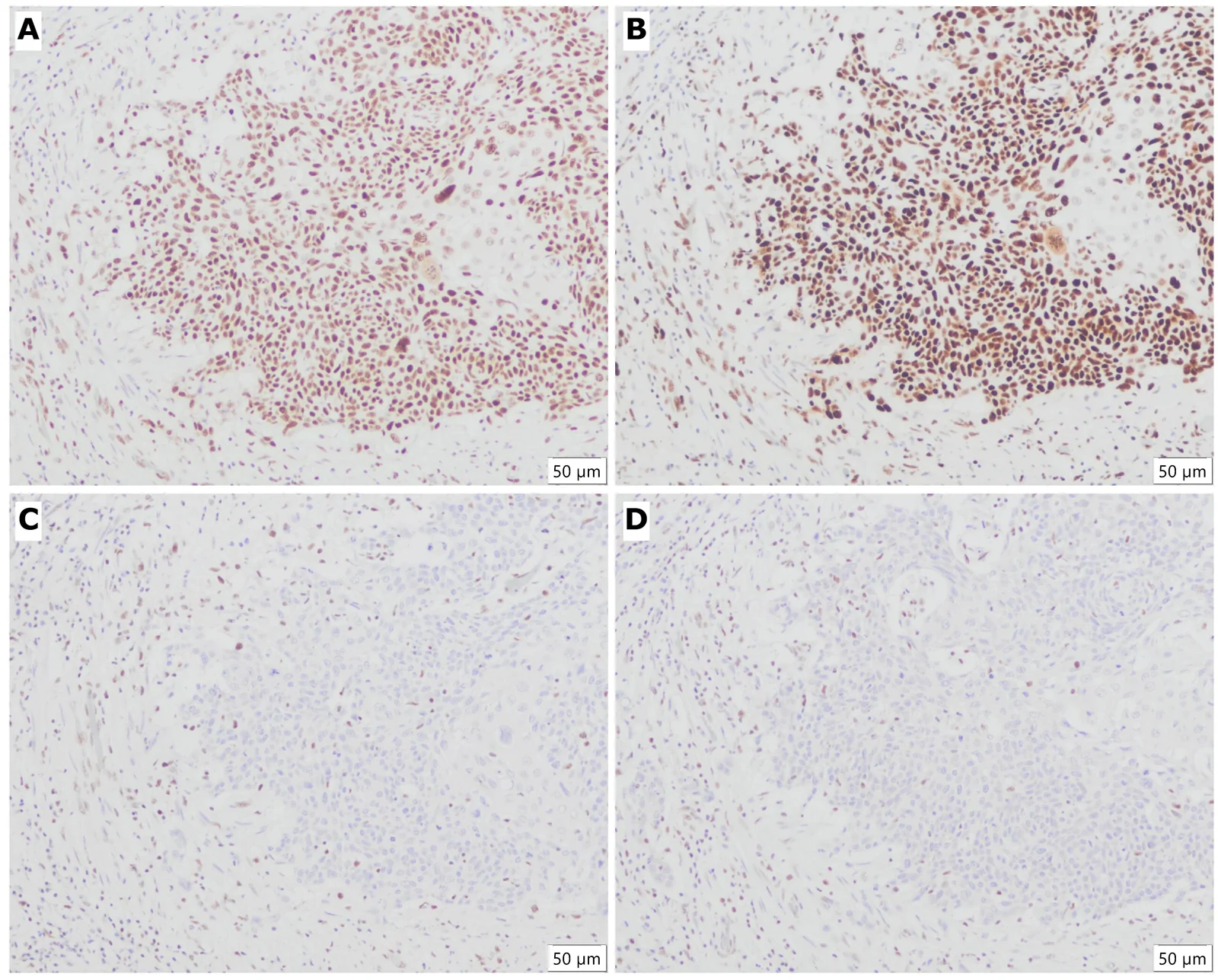
Micrographs of immunohistochemistry staining of mismatch repair (MMR) proteins in the tumor cells. **(A)** Retained nuclear expression of MSH2 (200x); **(B)** Retained nuclear expression of MSH6 (200x); **(C)** Loss of nuclear expression of MLH1(200x); **(D)** Loss of nuclear expression of PMS2 (200x).

In this regard, we obtained 2.6% patients being MSI-H. In the primary tumor, one patient was inconclusive for MSI and was pMMR in IHC, the two others were MSI-H and showed dMMR in IHC (**Figure 4**). In the metastases samples of these same three patients, all were MSI-H by molecular analysis and with loss of heterodimer MLH1/PMS2 by IHC (**Table 3**).

**Table 3:** Description of the 03 patients who had MSI and IHC results.

Patient	Clinical feature	Staging (AJCC)	Status	Site	MSI status	MLH1*	PMS2*	MSH2*	MSH6*	p16 status*
ID 266	49 yo, SCC, Grade 2, treated with adjuvant CR	pT3pN3	Alive at last folllow-up (48mo)	Primary	High	Loss	Loss	Proficient	Proficient	Positive
				Metastasis	High	Loss	Loss	Proficient	Proficient	
ID 84	73 yo, SCC, Grade 2, treated with adjuvant chemotherapy	pT2pN3	Deceased (OS: 04mo)	Primary	High	Loss	Loss	Proficient	Proficient	Positive
				Metastasis	High	Loss	Loss	Proficient	Proficient	
ID 80	71 yo, SCC, Grade 3, treated with adjuvant chemotherapy	pT1apN2	Deceased (OS: 12mo)	Primary	Inconclusive	Proficient	Proficient	Proficient	Proficient	Positive
				Metastasis	High	Loss	Loss	Proficient	Proficient	

AJCC: American Joint Committee on Cancer; CR: chemo-radiation therapy; SCC: squamous cell carcinoma; IHC: immunohistochemistry; OS overall survival; * Expression by immunohistochemistry.

The median survival between MSI-H and MSS was not significant (p 0.421), with worse outcomes for MSI-H 0.9 years vs. 11.2 years.

## 4 Discussion

Brazil has one of the highest incidences of penile cancer worldwide. In the present study, we report for the first time the concordance of MSI status between paired primary penile tumors and their corresponding lymph node metastases, demonstrating complete concordance in all MSI-H cases. We also observed that all MSI-H tumors were p16-positive, suggesting a possible association with HPV-related carcinogenesis.

Our cohort is representative of patients with penile squamous cell carcinoma treated in Brazilian referral centers, with a median age of 55 years, a predominance of uncircumcised men, advanced-stage disease, and clinicopathological characteristics comparable to those reported in other national series (26). Consistent with previous Brazilian studies, overall survival was similar to published data, and nodal involvement remained the strongest prognostic factor, reinforcing its central role in disease progression and survival (26, 27). These findings support the representativeness of our cohort and provide an appropriate clinical context for the interpretation of the molecular analyses.

All patients in our study underwent lymphadenectomy, either inguinal or pelvic, with a noteworthy pN0 frequency of 33.6%. This finding highlights the well-known clinical dilemma regarding the indication of lymphadenectomy, since only half of clinically positive lymph nodes harbor pathologically confirmed metastases (28). Conversely, other data suggest that up to 36.9% of cN0 cases may represent false positives (29). Although the prognostic value of nodal disease (N+) is well established, clinical evaluation alone does not appear accurate enough to determine surgical management, as corroborated by our findings.

The identification of p16 expression has been incorporated into WHO classifications as a validated surrogate biomarker of HPV-driven carcinogenesis. In our study, 22.8% of patients were p16-positive, and this subgroup demonstrated a 55% relative risk reduction of death. The Brazilian state of Maranhão, which has the world’s highest incidence of penile cancer, shows a frequency of 62% HPV-associated PeCA but also a high prevalence of other risk factors, such as poor genital hygiene (73%) and zoophilia practices (60%) (30). Olesen et al. evaluated 2,295 patients in a systematic review and meta-analysis and found 41.6% p16 positivity globally, though prevalence varies widely (13). Another systematic review and meta-analysis of 649 patients, 47% of whom were p16-positive, demonstrated better disease-specific survival among HPV- and p16-positive patients (31). Furthermore, a prospective phase II trial using atezolizumab with or without radiotherapy for PeCA reported that 43.8% of patients were p16-positive and experienced better survival outcomes compared to p16-negative patients (32). These data are aligned with our findings, underscoring the prognostic significance of HPV-associated PeCA in both chemotherapy and emerging immunotherapy contexts.

The low frequency of MSI-H observed in our cohort is consistent with findings in other squamous cell carcinomas, including vulvar SCC and head and neck SCC, where mismatch repair deficiency and MSI-H are uncommon molecular events (33, 34). MSI-H was identified in only 2.6% of cases, indicating that defective mismatch repair is a rare event in penile SCC. To our knowledge, this is the first study to compare MSI status between paired primary penile tumors and corresponding lymph node metastases; therefore, no previous studies in penile cancer are available for direct comparison. We observed complete concordance of MSI status between the primary tumors and matched metastases. Similar high concordance has been reported in colorectal cancer, where MSI/MMR status is generally preserved between primary tumors and metastatic sites (35, 36). A recent meta-analysis further demonstrated that MSI is among the most stable predictive biomarkers of immunotherapy across primary and metastatic tumors (37). Immunohistochemistry confirmed the molecular findings, with loss of the MLH1/PMS2 heterodimer in all MSI-H metastatic samples and in the two evaluable primary tumors, consistent with the expected MMR-deficient phenotype (23). All MSI-H tumors were also p16-positive. However, the small number of MSI-H cases precludes conclusions regarding an association between HPV-associated carcinogenesis and mismatch repair deficiency.

The association between genomic instability and tumor immunogenicity is well established, particularly in tumors with high TMB and MSI-H status, which derive greater benefit from immune checkpoint blockade. Although immunotherapy has recently been investigated in advanced penile cancer, MSI status has not been prospectively evaluated as a predictive biomarker in clinical trials (38). Necchi et al. reported that approximately 15% of penile SCCs harbor high TMB (19), while the HERCULES trial demonstrated substantially higher objective response rates among patients with TMB-high tumors treated with pembrolizumab plus platinum-based chemotherapy (75% vs. 36.4%) (38). Because MSI-H represents one of the biological mechanisms underlying high TMB, whereas other alterations such as POLE mutations may also generate hypermutated tumors (39), comprehensive molecular profiling may help identify the subset of patients most likely to benefit from immunotherapy. Although MSI-H appears to be rare in penile SCC, its established predictive value across solid tumors supports routine consideration of MSI testing, particularly in patients with advanced or treatment-refractory disease.

Our study comprises a relevant cohort considering the rarity of the PeCA, and it appears representative given the similarity of sample characteristics and clinical outcomes with the literature. We employed validated MSI-H testing methodology in the Brazilian population, and the paired analysis of primary tumors and nodal metastases provided novel insights into PeCA carcinogenesis and progression, as well as hypothesis-generating findings for future research.

Despite our findings, the study also has some limitations, including its relatively small sample size, single-institution data, and retrospective nature, which may introduce selection biases. Additionally, variability in HPV prevalence across Brazilian population may limit the generalizability of our findings, and HPV status was inferred solely by p16 immunohistochemistry, an accepted WHO surrogate marker that may misclassify HPV status in the absence of HPV DNA or RNA testing.

## 5 Conclusion

MSI-H is rare in penile cancers and is associated with p16-positive tumors. We observed a 55% reduced relative risk of death in p16 positive patients. More comprehensive, multicenter studies across different local cultures are needed to confirm our results.

## Data Availability

The datasets generated and/or analyzed during the current study are not publicly available due to ethical, legal, and privacy-related restrictions. De-identified data may be made available from the corresponding author upon reasonable request and subject to approval by the institutional ethics committee.

## References

[1] Favorito LA Nardi AC Ronalsa M Zequi SC Sampaio FJB Glina S . Epidemiologic study on penile cancer in Brazil. Int Braz J Urol. (2008) 34:587–91.discussion 591-3. doi: 10.1590/s1677-55382008000500007 18986562

[2] Wabinga HR Parkin DM Wabwire-Mangen F Nambooze S . Trends in cancer incidence in Kyadondo County, Uganda, 1960-1997. Br J Cancer. (2000) 82:1585–92. doi: 10.1054/bjoc.1999.1071 10789729 PMC2363394

[3] Bray F Laversanne M Sung H Ferlay J Siegel RL Soerjomataram I . Global cancer statistics 2022: GLOBOCAN estimates of incidence and mortality worldwide for 36 cancers in 185 countries. CA Cancer J Clin. (2024) 74:229–63. doi: 10.3322/caac.21834 38572751

[4] Pow-Sang MR Ferreira U Pow-Sang JM Nardi AC Destefano V . Epidemiology and natural history of penile cancer. Urology. (2010) 76:S2–6. doi: 10.1016/j.urology.2010.03.003 20691882

[5] Maden C Sherman KJ Beckmann AM Hislop TG Teh CZ Ashley RL . History of circumcision, medical conditions, and sexual activity and risk of penile cancer. J Natl Cancer Inst. (1993) 85:19–24. doi: 10.1093/jnci/85.1.19 8380060

[6] Engels EA Pfeiffer RM Goedert JJ Virgo P McNeel TS Scoppa SM . Trends in cancer risk among people with AIDS in the United States 1980-2002. AIDS. (2006) 20:1645–54. doi: 10.1097/01.aids.0000238411.75324.59 16868446

[7] Barbagli G Palminteri E Mirri F Guazzoni G Turini D Lazzeri M . Penile carcinoma in patients with genital lichen sclerosus: a multicenter survey. J Urol. (2006) 175:1359–63. doi: 10.1016/S0022-5347(05)00735-4 16515998

[8] Stern RS . Genital tumors among men with psoriasis exposed to psoralens and ultraviolet A radiation (PUVA) and ultraviolet B radiation. The Photochemotherapy Follow-up Study. N Engl J Med. (1990) 322:1093–7. doi: 10.1056/NEJM199004193221601 2320078

[9] Moch H Amin MB Berney DM Compérat EM Gill AJ Hartmann A . The 2022 world health organization classification of tumours of the urinary system and male genital organs-part A: renal, penile, and testicular tumours. Eur Urol. (2022) 82:458–68. doi: 10.1016/j.eururo.2022.06.016 35853783

[10] Marconnet L Rigaud J Bouchot O . Long-term followup of penile carcinoma with high risk for lymph node invasion treated with inguinal lymphadenectomy. J Urol. (2010) 183:2227–32. doi: 10.1016/j.juro.2010.02.025 20399455

[11] Ficarra V Akduman B Bouchot O Palou J Tobias-MaChado M . Prognostic factors in penile cancer. Urology. (2010) 76:S66–73. doi: 10.1016/j.urology.2010.04.008 20691887

[12] Mustasam A Parza K Ionescu F Gullapalli K Paravathaneni M Kim Y . The prognostic role of HPV or p16INK4a status in penile squamous cell carcinoma: A meta-analysis. J Natl Compr Canc Netw. (2025) 23. doi: 10.6004/jnccn.2024.7078 39752880

[13] Olesen TB Sand FL Rasmussen CL Albieri V Toft BG Norrild B . Prevalence of human papillomavirus DNA and p16INK4a in penile cancer and penile intraepithelial neoplasia: a systematic review and meta-analysis. Lancet Oncol. (2019) 20:145–58. doi: 10.1016/S1470-2045(18)30682-X 30573285

[14] Bordon Y . Immunotherapy: checkpoint parley. Nat Rev Cancer. (2015) 15:3. doi: 10.1038/nrc3880 25503072

[15] Wood RD Mitchell M Sgouros J Lindahl T . Human DNA repair genes. Sci (1979). (2001) 291:1284–9. doi: 10.1126/SCIENCE.1056154 11181991

[16] Liu B Nicolaides NC Markowitz S Willson JKV Parsons RE Jen J . Mismatch repair gene defects in sporadic colorectal cancers with microsatellite instability. Nat Genet. (1995) 9:48–55. doi: 10.1038/NG0195-48 7704024

[17] Palmeri M Mehnert J Silk AW Jabbour SK Ganesan S Popli P . Real-world application of tumor mutational burden-high (TMB-high) and microsatellite instability (MSI) confirms their utility as immunotherapy biomarkers. ESMO Open. (2022) 7:100336. doi: 10.1016/j.esmoop.2021.100336 34953399 PMC8717431

[18] Montella M Sabetta R Ronchi A De Sio M Arcaniolo D De Vita F . Immunotherapy in penile squamous cell carcinoma: present or future? Multi-target analysis of programmed cell death ligand 1 expression and microsatellite instability. Front Med (Lausanne). (2022) 9:874213. doi: 10.3389/fmed.2022.874213 35592855 PMC9113025

[19] Necchi A Spiess PE Costa de Padua T Li R Grivas P Huang RSP . Genomic profiles and clinical outcomes of penile squamous cell carcinoma with elevated tumor mutational burden. JAMA Netw Open. (2023) 6:e2348002. doi: 10.1001/jamanetworkopen.2023.48002 38150257 PMC10753400

[20] Harris PA Taylor R Thielke R Payne J Gonzalez N Conde JG . Research electronic data capture (REDCap)-A metadata-driven methodology and workflow process for providing translational research informatics support. J BioMed Inform. (2009) 42:377–81. doi: 10.1016/J.JBI.2008.08.010 18929686 PMC2700030

[21] Moch H Cubilla AL Humphrey PA Reuter VE Ulbright TM . The 2016 WHO classification of tumours of the urinary system and male genital organs—Part A: renal, penile, and testicular tumours. Eur Urol. (2016) 70:93–105. doi: 10.1016/J.EURURO.2016.02.029 26935559

[22] Chahoud J Zacharias NM Pham R Qiao W Guo M Lu X . Prognostic significance of p16 and its relationship with human papillomavirus status in patients with penile squamous cell carcinoma: results of 5 years follow-up. Cancers (Basel). (2022) 14:6024. doi: 10.3390/CANCERS14246024/S1 36551510 PMC9775956

[23] Joost P Veurink N Holck S Klarskov L Bojesen A Harbo M . Heterogenous mismatch-repair status in colorectal cancer. Diagn Pathol. (2014) 9. doi: 10.1186/1746-1596-9-126 24968821 PMC4074838

[24] Campanella NC Berardinelli GN Scapulatempo-Neto C Viana D Palmero EI Pereira R . Optimization of a pentaplex panel for MSI analysis without control DNA in a Brazilian population: correlation with ancestry markers. Eur J Hum Genet. (2014) 22:875–80. doi: 10.1038/EJHG.2013.256 24193342 PMC4060109

[25] Berardinelli GN Scapulatempo-Neto C Durães R Antônio de Oliveira M Guimarães D Reis RM . Advantage of HSP110 (T17) marker inclusion for microsatellite instability (MSI) detection in colorectal cancer patients. Oncotarget. (2018) 9:28691–701. doi: 10.18632/oncotarget.25611 29983889 PMC6033349

[26] Silva T Ximenes ÉGP Santos YS Araújo RJ Macedo E Medeiros K . Epidemiological study of penile cancer in a northeastern state - Brazil. Rev Col Bras Cir. (2023) 50:e20233586. doi: 10.1590/0100-6991e-20233586-en 37971116 PMC10626489

[27] Tappero S Piccinelli M Barletta F Panunzio A Cano Garcia C Incesu RB . Effect of inguinal lymph node dissection in lymph node negative patients with squamous cell carcinoma of the penis. World J Urol. (2023) 41:119–25. doi: 10.1007/S00345-022-04184-Z 36239810

[28] Koifman L . Risk factors for inguinal lymph node metastasis in patients with penile cancer. Int Braz J Urol. (2022) 48:314–5. doi: 10.1590/S1677-5538.IBJU.2021.0613.1 35170893 PMC8932034

[29] Maciel CM MaChado RD Morini MA Mattos PAL dos Reis R dos Reis RB . External validation of nomogram to predict inguinal lymph node metastasis in patients with penile cancer and clinically negative lymph nodes. Int Braz J Urol. (2019) 45:671–8. doi: 10.1590/S1677-5538.IBJU.2018.0756 31136111 PMC6837607

[30] Vieira CB Feitoza L Pinho J Teixeira-Júnior A Lages J Calixto J . Profile of patients with penile cancer in the region with the highest worldwide incidence. Sci Rep. (2020) 10:2965. doi: 10.1038/s41598-020-59831-5 32076037 PMC7031540

[31] Sand FL Rasmussen CL Frederiksen MH Andersen KK Kjaer SK . Prognostic significance of HPV and p16 status in men diagnosed with penile cancer: A systematic review and meta-analysis. Cancer Epidemiol Biomarkers Prev. (2018) 27:1123–32. doi: 10.1158/1055-9965.EPI-18-0322 29987099

[32] De Vries HM Rafael TS Gil-Jimenez A De Feijter JM Bekers E Van Der Laan E . Atezolizumab with or without radiotherapy for advanced squamous cell carcinoma of the penis (The PERICLES study): A phase II trial. J Clin Oncol. (2023) 41:4872–80. doi: 10.1200/JCO.22.02894/ASSET/952C46E9-A10D-4602-B6A1-46D451CFDB12/ASSETS/IMAGES/LARGE/JCO.22.02894APP3.JPG 37487169

[33] Zuo C Zhang H Spencer HJ Vural E Suen JY Schichman SA . Increased microsatellite instability and epigenetic inactivation of the hMLH1 gene in head and neck squamous cell carcinoma. Otolaryngol Head Neck Surg. (2009) 141:484–90. doi: 10.1016/j.otohns.2009.07.007 19786217

[34] Carreras-Dieguez N Guerrero J Rodrigo-Calvo MT Ribera-Cortada I Trias I Jares P . Molecular landscape of vulvar squamous cell carcinoma. Int J Mol Sci. (2021) 22. doi: 10.3390/ijms22137069 34209172 PMC8269046

[35] Haraldsdottir S Roth R Pearlman R Hampel H Arnold CA Frankel WL . Mismatch repair deficiency concordance between primary colorectal cancer and corresponding metastasis. Fam Cancer. (2016) 15:253–60. doi: 10.1007/s10689-015-9856-2 26666765

[36] He WZ Hu WM Wang F Rong YM Yang L Xie QK . Comparison of mismatch repair status between primary and matched metastatic sites in patients with colorectal cancer. J Natl Compr Canc Netw. (2019) 17:1174–83. doi: 10.6004/jnccn.2019.7308 31590148

[37] Zou Y Hu X Zheng S Yang A Li X Tang H . Discordance of immunotherapy response predictive biomarkers between primary lesions and paired metastases in tumours: A systematic review and meta-analysis. EBioMedicine. (2021) 63:103137. doi: 10.1016/j.ebiom.2020.103137 33310681 PMC7736926

[38] Cotait Maluf F Trindade K Preto D De Almeida Luz M Medeiros Milhomem Beato P Assed Bastos D . Pembrolizumab plus platinum-based chemotherapy for patients with advanced penile cancer: the nonrandomized HERCULES (LACOG 0218) clinical trial. JAMA Oncol. (2025) 11:1314–20. doi: 10.1001/jamaoncol.2025.3266 40965911 PMC12447286

[39] Zhao Z Li W Zhang X Ge M Song C . Correlation between TMB and MSI in patients with solid tumors. J Clin Oncol. (2020) 38:e15169–9. doi: 10.1200/JCO.2020.38.15_suppl.e15169 42148471

